# Protective effects of avocado peel and seed extracts against UVB-damaged fibroblasts for the development of an anti-photoaging nanoemulgel

**DOI:** 10.1038/s41598-025-13679-9

**Published:** 2025-07-31

**Authors:** Suradwadee Thungmungmee, Nakuntwalai Wisidsri

**Affiliations:** Faculty of Integrative Medicine, Rajamangala University of Technology Thanyaburi, Thanyaburi, Pathum Thani 12130 Thailand

**Keywords:** Avocado, Photoaging, Antioxidant, UVB, Nanoemulgel, Biotechnology, Cell biology, Molecular biology

## Abstract

This study investigates the anti-photoaging potential of ethanolic extracts from avocado peel and seed and their application in a nanoemulgel formulation. Both extracts demonstrated high phenolic content and strong antioxidant activity, with DPPH IC_50_ values of 57.05 ± 1.65 µg/ml (peel) and 52.44 ± 0.32 µg/ml (seed). In UVB-irradiated human dermal fibroblasts (BJ cells), the extracts (12.5–50 µg/ml) significantly reduced intracellular ROS, decreased MMP-1 and pro-inflammatory cytokines (TNF-α, IL-1β, IL-6), and restored collagen levels. A nanoemulgel was developed using the phase inversion composition (PIC) method, with avocado oil, isononyl isononanoate, Tween 60, and polyglyceryl-3 diisostearate, which resulted in nanoemulsions (NEs) with an average droplet size of 84.45 ± 5.45 nm and a zeta potential of −69.94 ± 5.66 mV. Incorporation of 0.5% Aqupec HV-505E and avocado extracts produced stable, light green gel. The resulting avocado extract-loaded nanoemulgel (ANE) demonstrated antioxidant activity, cytocompatibility with BJ cells, and stability in terms of color, pH, and viscosity. These findings reveal the anti-aging potential of avocado peel and seed extracts in preserving effectiveness within nanoemulgel for skin applications.

**Supplementary Information:**

The online version contains supplementary material available at 10.1038/s41598-025-13679-9.

## Introduction

Skin is the first barrier protecting the body against environmental stressors. The high-risk environment can cause an alteration of skin integrity. Among the high-risk stressors, ultraviolet (UV) is documented as a critical harmful factor to damage the skin^1^. UV wavelengths lie in the range of 100–400 nm and are further subdivided into UVC (100–280 nm), UVB (280–315 nm), and UVA (315–400 nm). Compared with UVA, UVB is significantly highly energic and carcinogenic^2^. Although the ozone layer can protect against UVB radiation penetration, the depletion of the ozone layer allows higher levels of UVB at the Earth’s surface. UVB damages the skin, causing sunburns, hyperpigmentation, photoaging, and photocarcinogenesis^3^.

The epidermis can mostly absorb UVB; however, UVB with high energic photons can penetrate through the upper part of the dermis^4^. In addition, UVB also induces skin cells to overproduce intracellular reactive oxygen species (ROS). Derogatory for cellular damaging effects, ROS include singlet oxygen^1^O_2_), superoxide anion (^•^O_2−_), hydroxyl radical (^•^HO), and the non-radical hydrogen peroxide (H_2_O_2_). ROS are the initial molecules that trigger several signaling transduction pathways involved in alternating skin integrity. Finally, the matrix metalloproteinases (MMPs) are induced to high production, including MMP-1, -2, -3, and − 9^5^. They are responsible for breaking down the collagen fibrils, including collagen type I, III, IV, and other ECM, such as elastin, proteoglycans, and laminins. In particular, MMP-1 is mainly for degrading dermal type I collagen, the most abundant structural protein in the dermis^6^ to cause a reduction in skin strength and elasticity, contributing to skin wrinkles, the main characteristic of photoaging^7^.

Avocado (*Persea americana* Mill.) is cultivated in tropical and subtropical climates worldwide, belonging to the Lauraceae family. It is widely used in Ayurveda for treating menorrhagia, hypertension, diarrhea, diabetes, and evidence-based phototherapy^8^. It is considered a superfood due to its rich nutritional profile, which includes vitamins, minerals, proteins, dietary fiber, unsaturated fatty acids, and a wide range of bioactive compounds such as carotenoids, hydroxybenzoic and hydroxycinnamic acids, procyanidins, tannins, and flavonoids^9^. Although the non-edible parts of the avocado, like peels and seeds, are often discarded. The avocado peels contain significant concentrations of bioactive compounds, including carotenoids, tocopherols, and various phenolic compounds^10,11^. In addition, avocado seeds have been shown the value nutrients and bioactive compounds as potential health benefits as well, including polysaccharides, proteins, lipids, vitamins, minerals, and phytochemicals such as acetogenin, catechin, epicatechin, and procyanidin B1^12,13^. Avocado peel and seed extracts have been shown to offer therapeutic benefits in combating various human degenerative diseases linked to reactive oxygen species (ROS) and oxidative stress. These benefits include prevention and protection against neurodegeneration, cardiovascular and gastrointestinal disorders, and the development of cancer^14–16^. Previous studies have shown the pharmacological effects of various parts of avocado extracts. Avocado leaf extract prepared with a hydroethanolic solvent (ethanol: water, 70:30 v/v) and formulated into a gel demonstrated antinociceptive and anti-inflammatory effects in a UVB-induced burn model in mice^17^. Moreover, avocado peel extract obtained through methanolic maceration and formulated into an ointment has been shown to enhance the healing of second-degree burn wounds in mice^18^. In the context of anti-skin aging, various studies have highlighted the benefits of avocado oil, attributed to its antioxidant properties and the presence of various bioactive compounds^19–22^. Consequently, avocado oil has been incorporated into topical formulations aimed at combating inflammation, skin damage, and aging^23–25^. These findings underscore the potential of avocado-derived ingredients in the development of functional foods, nutraceuticals, and pharmaceutical products. However, fundamental knowledge regarding the biological properties of avocado peel and seed extracts remains limited, particularly in the context of anti-skin aging applications.

Nanoemulgel, known as nanoemulsions (NEs) incorporated with gelling agents, are developed to address the limitations of NEs, such as low spreadability, difficulty in use, and reduced permeability^26,27^. Additionally, nanoemulgel is beneficial alternatives to traditional gel formulations for hydrophobic substances. Consequently, natural substance-loaded nanoemulgel has recently been used to improve therapeutic effectiveness and offer antioxidant, anti-inflammatory, and wound healing benefits^8^.

For these reasons, most prior work has not examined the effects of avocado peel and seed extracts in skin-targeted delivery systems or explored the molecular mechanisms involved in skin aging pathways. Our study addresses this gap by evaluating the anti-photoaging effects of avocado peel and seed extracts specifically the protective roles against UVB-induced skin cell damage providing mechanistic insight into their potential use in anti-aging skincare applications. Avocado peel and seed extracts were incorporated into nanoemulgel to create a novel anti-photoaging product. The study seeks to repurpose avocado waste for skincare, promoting environmental sustainability and advancements in cosmeceutical sciences.

## Materials and methods

### Chemicals and reagents

2,2-Diphenyl-1-picrylhydrazyl (DPPH), hydrogen peroxide (H_2_O_2_), dichlorofluorescin diacetate (DCFDA), resazurin, and vitamin C (Vit C) were purchased from Sigma-Aldrich, USA. The analytical grades of ethanol, hydrogen peroxide, dimethyl sulfoxide (DMSO), and methanol were purchased from RCI Labscan, Thailand. Dulbecco’s Modified Eagle’s Medium (DMEM), 0.25% Trypsin-EDTA, fetal bovine serum, 0.4% trypan blue, penicillin, and streptomycin were purchased from Gibco, USA. RIPA buffer, protease, phosphatase inhibitor cocktail, and horseradish peroxidase-conjugated secondary antibody were purchased from Abcam, USA. MMP-1 primary antibody and collagen Human COL1α1 (Collagen Type I Alpha 1) ELISA Kit were provided by Elabscience Biotechnology, USA. The cosmetic grade ingredients, including Tween 60, polyglyceryl-3 diisostearate, avocado oil, isononyl isononanoate, propylene glycol, triethanolamine, and phenoxyethanol were purchased from CHEME COSMETICS, Thailand. Aqupec HV-505E was provided by Sumitomo Seika Chemicals, Japan.

### Preparation of avocado peel and seed extracts

Ripe Hass avocados were purchased from the local market in Chiang Mai province, Thailand. The peels and seeds were separated from the pulp, thoroughly washed with water, sliced, and dried at 50 °C for 24 h. Once dried, the peels and seeds were ground into powder. The extraction method used to achieve high yields of phenolic and flavonoid compounds was slightly adapted based on the procedures described by Ratananikom and Preprayoon (2022) and Prommajak et al. (2014)^28,29^. Ten grams of this powder were dissolved in 200 ml of 70% ethanol and subjected to ultrasonic extraction using an Elmasonic S 300 H (Germany) at a frequency of 60 Hz and power of 1500 W. The extraction was carried out for 30 min at a controlled temperature of 50 °C. Following the extraction, the mixture was filtered, and the solvent was evaporated at 45 °C using a rotary evaporator. The resulting crude extracts from the avocado peel and seed were stored at -20 °C until further use.

### Determination of the total phenolic content

The total phenolic content (TPC) of avocado peel and seed extracts was determined by Folin–Ciocalteu assay, which was slightly modified from Paudel et al. (2018)^30^. Twenty µl of extract or ANE was mixed with 100 µl of 10%Folin-Ciocalteu’s reagent. The mixture was incubated at room temperature for 7 min. Then, 100 µl of 7.5% sodium carbonate was added and incubated at room temperature for 45 min. The mixture absorbance was measured using a Microplate reader CALIOstar (BMG LABTECH, Germany) at 765 nm. TPC was obtained from a calibration curve of standard gallic acid and was expressed in mg GAE/g dry extract or mg GAE/g ANE. The experiment was performed in triplicate.

### 2,2-diphenyl-1-picrylhydrazyl (DPPH) assay

The antioxidant activity of avocado peels and seed extracts was determined using a stable free radical DPPH assay. Briefly, the extracts at various concentrations of approximately 20 µl were mixed with 180 µl of 0.20 mM DPPH solution (in methanol). The mixture solutions were kept for 30 min without direct exposure to light. The absorbance was subsequently measured at 520 nm using a microplate reader^31^. Vitamin C (Vit C) and 0.2% DMSO were used as the positive and negative controls, respectively. The percentage of the DPPH scavenging activity and the IC_50_ were determined. The percentage of DPPH scavenging activity was calculated using the equation below:

$$\% {\text{DPPH scavenging activity}} = \left[ {\left( {{\text{OD}}_{{{\text{control}}}} - {\text{OD}}_{{{\text{sample}}}} } \right)/{\text{OD}}_{{{\text{control}}}} } \right] \times {\text{1}}00.$$

Where OD_control_ is the absorbance of control without DPPH and OD_sample_ is the absorbance of extract or positive control reacted with DPPH.

### Hydrogen peroxide (H_2_O_2_) scavenging assay

H_2_O_2_ can be changed to the hydroxyl radical (^•^HO), a highly reactive oxygen species, through the Fenton reaction, which is toxic to human cells. The peel and seed extracts of avocado were measured for their ability to neutralize H_2_O_2_. Briefly, 40 mM H_2_O_2_ solution was prepared in phosphate buffer (pH 7.4). Eighty µl of different concentrations of avocado peel or seed extracts (12.5–200 µg/ml) were mixed with H_2_O_2_ solution (720 µl). After 10 min of incubation, the absorbance was determined at 230 nm^31^. Vit C and phosphate buffer were used as positive and negative control, respectively. The percentage of the H_2_O_2_ scavenging and the IC_50_ were determined. The percentage of H_2_O_2_ scavenging activity was calculated by the following equation:

$$\% {\text{H}}_{{\text{2}}} {\text{O}}_{{\text{2}}} {\text{scavenging activity}} = \left[ {\left( {{\text{OD}}_{{{\text{control}}}} - {\text{OD}}_{{{\text{sample}}}} } \right)/{\text{OD}}_{{{\text{control}}}} } \right] \times {\text{1}}00.$$

Where OD_control_ is the absorbance of control without H_2_O_2_ and OD_sample_ is the absorbance of extract or positive control with H_2_O_2_.

### Cell culture

BJ cells (Lot No. 7005964, ATCC^®^-CRL-2522, USA), the human dermal fibroblasts were maintained in Dulbecco’s Modified Eagle Medium (DMEM) supplemented with 10% fetal bovine serum, 1% penicillin and streptomycin and incubated at 37 °C in 5% CO_2_ with humidified. During use, the cells were subcultured using 0.25% Trypsin-EDTA twice weekly. Cells with more than 90% viability determined by 0.4% trypan blue staining at 2 × 10^5^ cells/ml were used in this study.

### Resazurin reduction assay

The avocado peel and seed extracts were determined for their effects on the viability of BJ cells. The BJ cells at 2 × 10⁵ cells/ml were seeded into 96-well plates for 24 h and treated with various concentrations of avocado peel or seed extracts (6.25–100 µg/ml), vitamin C (100 µg/ml, positive control), or 0.2% DMSO (negative control) for another 24 h. The treated cells were then incubated with 50 µg/ml resazurin at 37 °C for 4 h. The absorbance was determined at 560 and 600 nm^32^. The percentage of cell viability was calculated compared to the 0.2% DMSO-treated cells (negative control) using the following equation:

$$\% {\text{Cell viability}} = \left[ {\left( {{\text{OD}}_{{{\text{56}}0}} - {\text{OD}}_{{{\text{6}}00}} } \right)_{{{\text{sample}}}} /\left( {{\text{OD}}_{{{\text{56}}0}} - {\text{OD}}_{{{\text{6}}00}} } \right)_{{{\text{control}}}} } \right] \times {\text{1}}00.$$

Where OD_control_ is the absorbance of 0.2%DMSO, the negative control, and OD_sample_ is the absorbance of extract or positive control.

### UVB irradiation procedure

The UVB irradiation procedure was followed a previously published study^32^. Briefly, BJ cells at 2 × 10^5^ cells/ml were maintained for 24 h with the culture medium in a 96-well plate for the next other experiments or 6-well plate for Western blot analysis. Following incubation, the cells were pre-treated with various concentrations of avocado peel or seed extracts (6.25–50 µg/ml), Vit C (100 µg/ml), or 0.2% DMSO for 24 h. After the pre-treatment period, the culture supernatants were removed, and the cells were washed twice with phosphate-buffered saline (PBS). A thin layer of PBS was added to cover the cells prior to UVB exposure. Irradiation was performed using a UVB lamp (Philips TL 20 W/01RS, 311 nm; Philips Lighting Holding B.V., The Netherlands) positioned 12 cm above the culture plates. The UVB intensity was measured using a UVB meter (Jedto Instruments, Thailand), and the exposure time was set to 1 min and 42 s to deliver a non-cytotoxic dose of 40 mJ/cm^2^.

### 2′,7′-dichlorofluorescin diacetate (DCFDA) assay

The ability of avocado peel and seed extracts to neutralize intracellular ROS was evaluated using the DCFDA assay. DCFDA is a cell-permeable fluorogenic dye that is oxidized by ROS to form the highly fluorescent compound 2′,7′-dichlorofluorescein (DCF). BJ cells at 2 × 10^5^ cells/ml were seeded in 96-well black clear-bottom plates for 24 h, pre-treated with avocado peel or seed extracts for another 24 h, and irradiated with 40 mJ/cm² of UVB. The treated- and irradiated- cells were then incubated with 30 µM of DCFDA at 37 °C for 30 min. After removing the supernatants and washing twice with PBS, the fluorescence intensities were determined at 485 mm excitation and 535 nm emission using a fluorescence microplate reader^32^.

### Western blot analysis

The levels of MMP-1 were determined by Western blot analysis. BJ cells at 2 × 10^5^ cells/ml were seeded in the 6-well plates for 24 h, pre-treated with the avocado peel or seed extracts for another 24 h. The treated cells were irradiated with 40 mJ/cm^2^ UVB and incubated for 24 h in culture medium. The total proteins were isolated by using RIPA buffer containing a protease inhibitor, and protein contents were determined by using NanoDrop Microvolume Spectrophotometers. Aliquoted 20 µg proteins were run on 10% SDS-PAGE gel and transferred to a nitrocellulose membrane. The membranes were blocked with 5% nonfat dry milk in Tris-HCL-based buffered saline with 0.1% Tween-20 (TBST) blocking solution for 1 h, washed with TBST 3 times for 5 min intervals, and incubated with the specific primary antibody against MMP-1 (1:1000) in the blocking solution overnight at 4 °C. Membranes were then washed 3 times with TBST for 5 min intervals before incubating with horseradish peroxidase (HRP)-conjugated secondary antibody in a blocking solution for 1 h, and washed with TBST thrice for 5 min interval. Immunoreactive bands were determined by using a chemiluminescence method by the CheBI Chemiluminescence Bioimaging Instrument. The images were acquired by NEOimage Program.

### Enzyme-linked immunosorbent assay (ELISA) for Human COL1α1

The supernatants were collected from previous experiments to determine the levels of collagen Human COL1α1 (Collagen Type I Alpha 1) ELISA Kit according to the manufacturing protocols. Briefly, 100 µl of standards and samples, were added to a 96-well plate which pre-coated with a specific antibody and incubated at 37 °C for 90 min. The liquid from each well was descended without washing. Immediately added 100 µl of biotinylated detection antibody and incubated for another hour at 37 °C. The wells were then washed three times with a wash buffer. Next, 100 µl of HRP-conjugated reagent was added and incubated for 30 min at 37 °C, followed by five more wash steps. Then, 90 µl of TMB substrate was added and incubated for 15 min in the dark at 37 °C. The reaction was stopped by adding 50 µl of stop solution, and absorbance was measured at 450 nm using a microplate reader. The COL1α1 concentration in each sample was determined using a standard curve.

### Enzyme-linked immunosorbent assay (ELISA) for pro-inflammatory cytokines

BJ cells were seed in 96 well-plate for 24 h and pre-treated with avocado peel or seed extracts for another 24 h and subsequently exposed to UVB irradiation. After 24 h of post-irradiation incubation, the culture supernatants were collected for cytokine analysis. Briefly, high-binding 96-well plates were coated overnight at 4 °C with capture-antibody specific to each cytokine. The coating solution was discarded, and the wells were blocked with a blocking buffer for 1 h at room temperature. After removal of the blocking buffer, either standards or supernatants were added to the wells and incubated for 2 h at room temperature. Following incubation, the wells were washed, and detection antibodies were added and incubated for 2 h at room temperature. After additional washes, poly-HRP-conjugated streptavidin was added and incubated for 30 min. Subsequently, TMB substrate solution was added to each well and allowed to react for 10 min in the dark. The enzymatic reactions were terminated by adding a stop solution, and absorbance was measured at 450 nm using a microplate reader. The levels of TNF-α, IL-1 and IL-6 were calculated from prepared standard curves.

### Nanoemulsion and nanoemulgel preparation

Nanoemulsions (NEs) were formulated as a PIC method. The weight ratios of surfactant (Tween 60) and co-surfactant (polyglyceryl-3 diisostearate) of 1:0 (NE1), 1:0.2 (NE2), and 1:0.4 (NE3) were varied (Supplementary Table S1). The HLB values of the mixture surfactant and co-surfactant in the range of 10–15 were used to prepare oil in water (o/w) nanoemulsion. The HLB values of the systems were calculated by following equation^33^:

$${\text{HLB system}} = {\text{HLB}}_{{\text{S}}} {\text{F}}_{{\text{S}}} + ~{\text{HLB}}_{{\text{C}}} {\text{F}}_{{\text{C}}}.$$

Where HLB_S_ and HLB_C_ are HLB values of Tween 60 and polyglyceryl-3 diisostearate, respectively, and F_S_ and F_C_ are the weight fractions of Tween 60 and polyglyceryl-3 diisostearate, respectively.

The mixture of surfactant and co-surfactant was mixed with the oil phase (avocado oil: isononyl isononanoate (1:10)) under magnetic stirrer at 800 rpm for 30 min. Deionized water was then stepwise added to the mixture by 0.5 ml/min under a magnetic stirrer at 800 rpm. After adding the full amount of water, the mixture was continued stirring for 20 min with a stirring rate of 500 rpm.

For nanoemulgel preparation, 0.50%w/w Aqupec HV-505E, a water-soluble cross-linked acrylic polymer, was dispersed into the mixture under a magnetic stirrer at 700 rpm. Triethanolamine was then added and stirred to form nanoemulgel. Then, phenoxyethanol was added. Finally, this formula is called base nanoemulgel (BNE). To perform avocado extracts-loaded nanoemulgel (ANE), the effective dose, 5% w/w of 2% avocado extracts dissolved in propylene glycol, was selected based on more than 10-fold of IC_50_ in DPPH assay^31^. The dissolved extract was mixed with BNE followed by adding triethanolamine. The formulations were set for 24 h before being characterized. The composition of BNE and ANE are shown in (Table 1).

**Table 1:** Composition of base nanoemulgel (BNE) and avocado extracts-loaded nanoemulgel (ANE) (%w/w).

Phase	Ingredients	INCI name	Function	Base nanoemulgel (BNE) (%w/w)	Avocado extracts-loaded nanoemulgel (ANE) (%w/w)
Oil	Polyglyceryl-3 Diisostearate	Polyglyceryl-3 Diisostearate	Co-surfactant	2.00	2.00
	Avocado oil: isononyl isononanoate (1:10)	*Persea gratissima* (Avocado) oil: Isononyl isononanoate	Emollient	10.00	10.00
Water	2% Avocado peel extract: avocado seed extract (ratio 2:0.5) dissolved in propylene glycol	–	Active ingredient	–	5.00
	Propylene glycol	Propylene glycol	Solvent	5.00	–
	Tween 60	Polysorbate 60	Surfactant	10.00	10.00
	Triethanolamine	Triethanolamine	pH adjuster	0.50	0.50
	Phenoxyethanol	Phenoxyethanol	Preservative	1.00	1.00
	Deionized water	Aqua	Solvent	72.00	72.00
Gel base	Aqupec HV-505E	Carbomer	Gelling agent	0.50	0.50

### Physicochemical characterization

The homogeneity and color of NEs and nanoemulgels were observed at room temperature. The phase separation was examined by centrifuge at 3000 rpm for 5 min. The droplet size, polydisperse index (PDI), and zeta potential of NEs were analyzed by Nanoparticle analyzer using dynamic light scattering (nanoPartica SZ-100V2 Series, HORIBA, Japan) at 25 °C with a scattering angle of 173º. NEs were diluted with deionized water 100 times before measurement. The smaller size and low PDI of each formulation were criteria for further nanoemulgel preparation. The pH value of nanoemulgel was measured using a pH meter at 25 °C. The viscosity of nanoemulgel was measured using a Brookfield viscometer with spindle no.4. All measurements were performed in triplicate.

### Cytotoxicity, total phenolic content (TPC) in vitro antioxidant, and stability

BNE and ANE were investigated for the effects on the viability of fibroblasts using resazurin reduction assay, measured TPC and the scavenging activity on DPPH radical, and studied for stability using a heating-cooling test for 7 cycles. In each cycle, the nanoemulgels were kept at 4 ± 1 °C for 24 h and 45 ± 1 °C for 24 h while maintaining a relative humidity of 75%. Then the physicochemical properties of the formulations, including homogeneity, color, phase separation, and pH, were compared to the initial time.

**Fig. 1:**
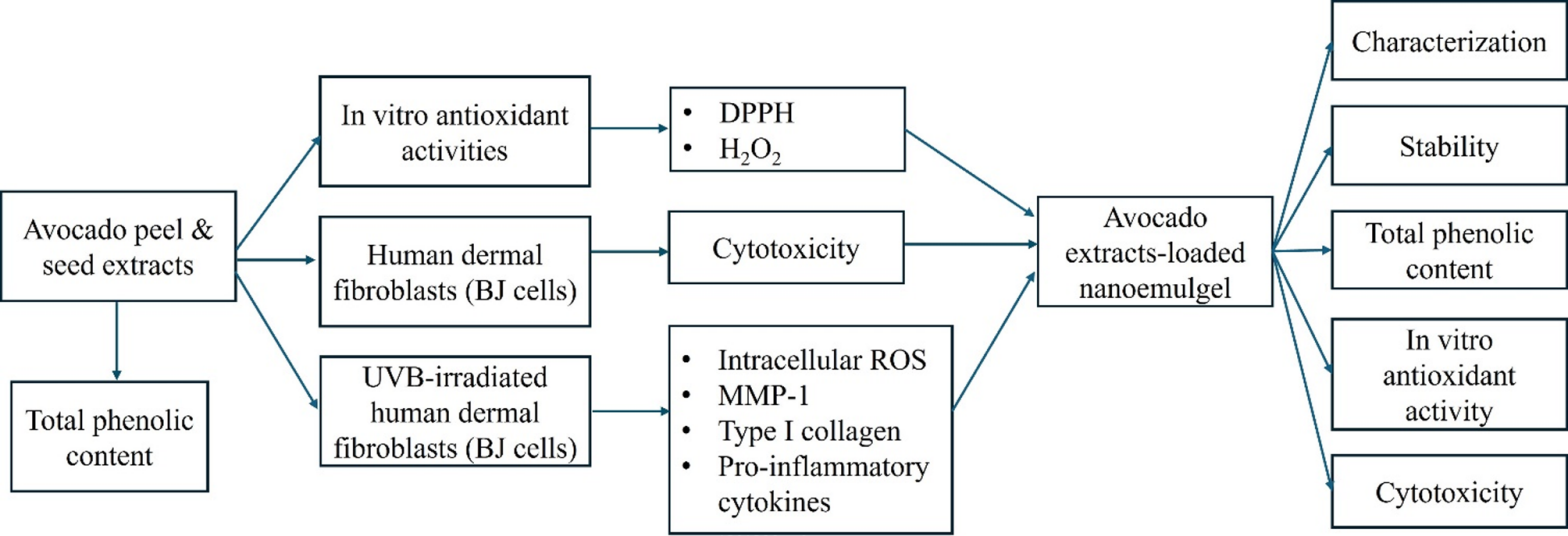
Article flow diagram.

The article flow diagram is shown in (Fig. 1).

### Statistical analysis

Results from three independent experiments were presented as mean with standard error of mean (mean ± SEM). The differences between the test compounds and the suitable control were compared using the ANOVA (analysis of variance) test, followed by Tukey’s post hoc test or Pair *t*-test. SPSS version 23 was used for statistical analysis. P-values less than 0.05 were considered to be statistically significant.

## Results

### Total phenolic content (TPC) of avocado peel and seed extracts

The content of phenolic compounds in avocado extracts ranged from 80.72 to 149.83 mg GAE/g dry extract. TPC of avocado seed extract had a greater value in avocado peel extract as shown in (Table 2).

**Table 2:** Total phenolic content of avocado Peel and seed extracts.

Sample	Total phenolic content (mg GAE/g dry extract)
Avocado seed extract	149.83 ± 9.57
Avocado peel extract	80.72 ± 10.05

### In vitro oxidant scavenging activities of avocado peel and seed extracts

The antioxidant activity of avocado peel and seed extracts was evaluated by their ability to scavenge DPPH radicals. The IC_50_ values for the peel and seed extracts were 57.05 ± 1.65 µg/ml and 52.44 ± 0.32 µg/ml, respectively (Table 3). Additionally, both extracts demonstrated the ability to scavenge H_2_O_2_ oxidants, with IC_50_ values of 123.23 ± 0.46 µg/ml for the peel extract and 73.80 ± 0.30 µg/ml for the seed extract. Vit C also showed DPPH and H_2_O_2_ scavenging activities which the IC_50_ values of 6.42 ± 0.05 and 149.57 ± 0.20 µg/ml, respectively (Table 3).

**Table 3:** DPPH and H_2_O_2_ scavenging activities of avocado Peel and seed extracts.

Compounds	IC_50_ (µg/ml)	
	DPPH	H_2_O_2_
Avocado peel extract	57.05 ± 1.65	123.23 ± 0.46
Avocado seed extract	52.44 ± 0.32	73.80 ± 0.30
Vit C	6.42 ± 0.05	149.57 ± 0.20

### Effects of avocado peel and seed extracts on the viability of BJ cells

**Fig. 2:**
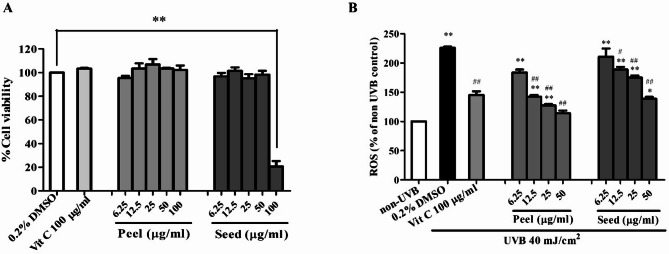
(**A**) Effects of avocado peel and seed extracts on the viability of BJ cells. The cells were treated with various concentrations of avocado peel and seed extracts at 6.25–100 µg/ml for 24 h. The viability of the treated cells was determined by resazurin reduction assay. (**B**) Effects of avocado peel and seed extracts on the intracellular ROS level in UVB-irradiated BJ cells. The cells were treated with avocado peel and seed extracts at 6.25–50 µg/ml or Vit C for 24 h, irradiated with 40 mJ/cm^2^ UVB, maintained in the culture medium for 30 min, and finally determined intracellular ROS levels using DCFDA assay. The results are presented as the mean ± SEM of three independent experiments. The statistical significance of differences was evaluated by one-way ANOVA followed by Tukey’s test. ^**^p < 0.01 compared to the non-irradiated control; ^#^*p* < 0.05, ^##^*p* < 0.01 compared to the UVB-irradiated control.

The viability of avocado peel extracts-treated BJ cells was similar to those of untreated cells by 97.10 ± 2.99, 103.43 ± 7.64, 103.12 ± 7.97, and 103.43 ± 1.21% in treated with avocado peel 6.25, 12.5, 25, and 50 µg/ml, respectively. Similar to the peel extracts, the viability of avocado peel extracts-treated BJ cells were 96.83 ± 5.12, 101.52 ± 5.06, 95.21 ± 5.89, and 98.85 ± 5.61% in treated with avocado seed 6.25, 12.5, 25, and 50 µg/ml, respectively. However, a decrease in BJ cell viability was observed when the concentration of the seed extract was increased to 100 µg/ml. Based on these findings, avocado peel and seed extracts at concentrations of 12.5–50 µg/ml were selected for subsequent experiments. (Fig. 2A).

### Intracellular ROS scavenging potency of avocado peel and seed extracts in UVB-irradiated BJ cells

The intracellular ROS-scavenging activity of avocado peel and seed extracts was evaluated at concentrations of 12.5–50 µg/ml. UVB was the immediate cause of inducing intracellular ROS of fibroblast cells. After UVB irradiation, BJ cells produced higher levels of intracellular ROS by 327.43 ± 6.28% compared to non-irradiated cells. The avocado peel and seed extracts could significantly decrease these effects by reducing these ROS in UVB-irradiated cells. The results revealed significantly decreasing ROS levels in 12.5–50 µg/ml concentrations of avocado peel and seed extracts (Fig. 2B). These protective results were also found in Vit C 100 µg/ml, the well-known antioxidant used as a positive control.

### Reduction of UVB-induced MMP-1 expression and anti-collagenolytic activities of avocado peel and seed extracts

**Fig. 3:**
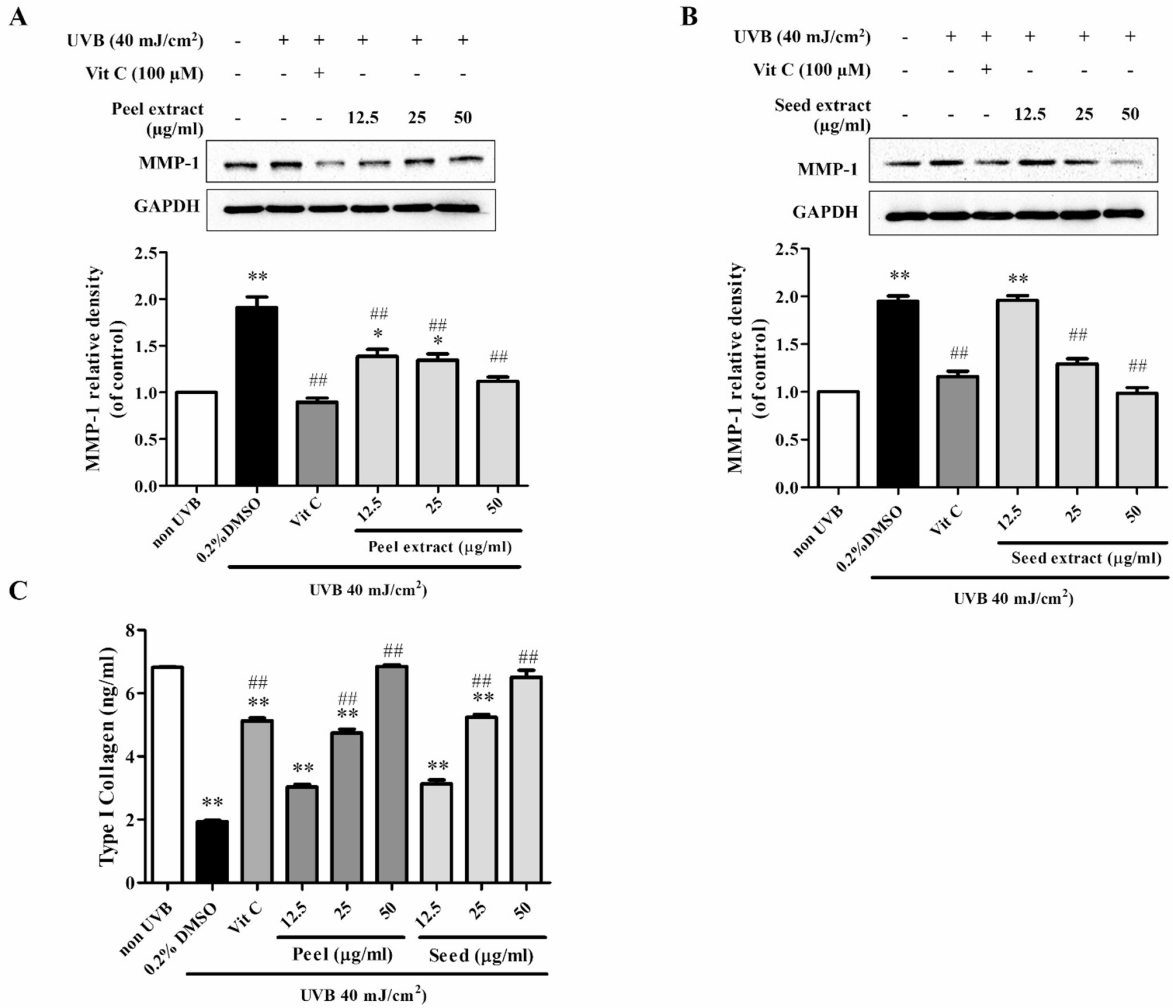
Effects of avocado peel and seed extracts on MMP-1 and type I collagen levels in UVB-irradiated BJ cells. The cells were pre-treated with 12.5–50 µg/ml of the extracts, irradiated with 40 mJ/cm^2^ UVB, maintained in the culture medium for 24 h, and determined the levels of MMP-1 proteins by Western blot analysis in (**A**) avocado peel extract and (**B**) avocado seed extract, and (**C**) determined the collagen content in avocado peel and seed extracts-treated cells in the supernatants by using ELISA after UVB irradiation 24 h. The results are presented as the mean ± SEM of three independent experiments. The statistical significance of differences was evaluated by one-way ANOVA followed by Tukey’s test. ^**^p < 0.01 compared to the non-irradiated control; ^#^*p* < 0.05, ^##^*p* < 0.01 compared to the UVB-irradiated control.

The protective effects of avocado peel and seed extracts on UVB-induced MMP-1 production in BJ fibroblasts were evaluated after 24 h of UVB irradiation. UVB exposure significantly increased MMP-1 levels about 2 folds in BJ cells compared to non-irradiated cells. Avocado peel extracts at 12.5 to 50 µg/ml concentrations significantly reduced MMP-1 levels to 1.39 ± 0.07-fold, 1.35 ± 0.07-fold, and 1.12 ± 0.05-fold, respectively. Similarly, avocado seed extracts at 25 and 50 µg/ml demonstrated protective effects by reducing MMP-1 levels to 1.29 ± 0.05-fold and 0.98 ± 0.06-fold, respectively (Fig. 3A,B).

Subsequently, collagen levels were assessed. The findings revealed that treatment with avocado peel or seed extracts reversed the effects of UVB in treated cells. Non-irradiated fibroblast cells produced collagen at approximately 6.82 ± 0.05 ng/ml. After UVB irradiation, collagen levels of fibroblasts decreased to 0.98 ± 0.01 ng/ml. Pre-treatment with avocado peel or seed extracts effectively restored collagen levels to values comparable to those of non-irradiated cells. Specifically, avocado peel extract increased collagen levels to 4.98 ± 0.01 ng/ml and 6.75 ± 0.01 ng/ml at concentrations of 25 and 50 µg/ml, respectively. Likewise, avocado seed extract elevated collagen levels to 5.11 ± 0.01 ng/ml and 6.18 ± 0.26 ng/ml at 25 and 50 µg/ml concentrations, respectively (Fig. 3C).

### Anti-inflammatory activities of avocado peel and seed extracts

**Fig. 4:**
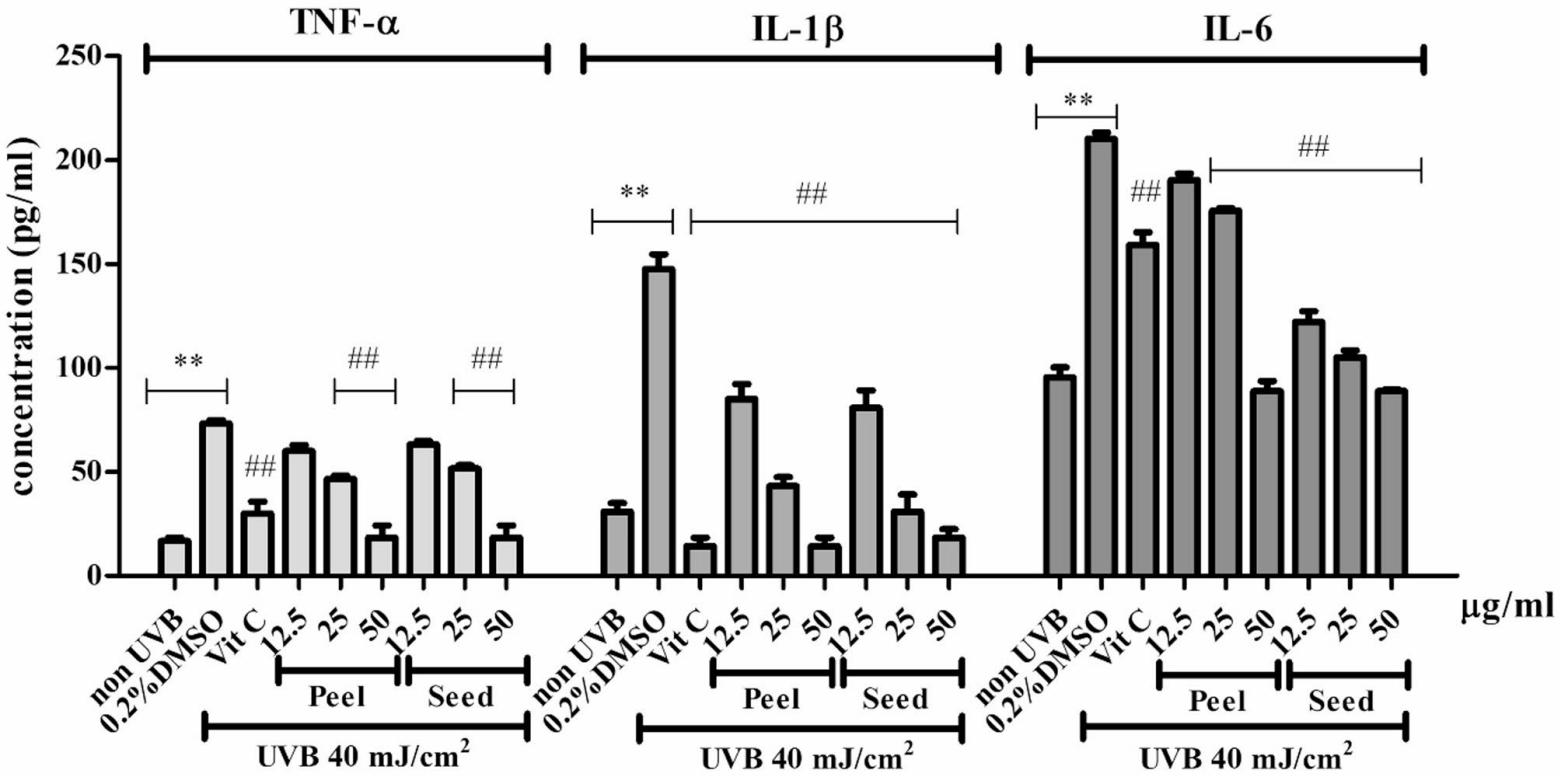
Effects of avocado peel and seed extracts on pro-inflammatory cytokines in UVB-irradiated BJ cells. The cells were pre-treated with 12.5–50 µg/ml of the extracts, irradiated with 40 mJ/cm^2^ UVB, maintained in the culture medium for 24 h, and collected supernatant for determining the levels of TNF-α, IL-1β, and IL-6 by ELISA. The statistical significance of differences was evaluated by one-way ANOVA followed by Tukey’s test. ^**^p < 0.01 compared to the non-irradiated control; ^##^*p* < 0.01 compared to the UVB-irradiated control.

Pro-inflammatory cytokines, including TNF-α, IL-1β, and IL-6 were determined by ELISA kit for each cytokine. Normally, BJ cells produce pro-inflammatory cytokines, at low level. After UVB-irradiation, the levels of these cytokines significantly increase. However, pre-treatment with avocado peel and seed extracts at 25 and 50 µg/ml and Vit C at 100 µg/ml completely reduced these pro-inflammatory cytokine levels (Fig. 4).

### Physicochemical characterization

**Fig. 5:**
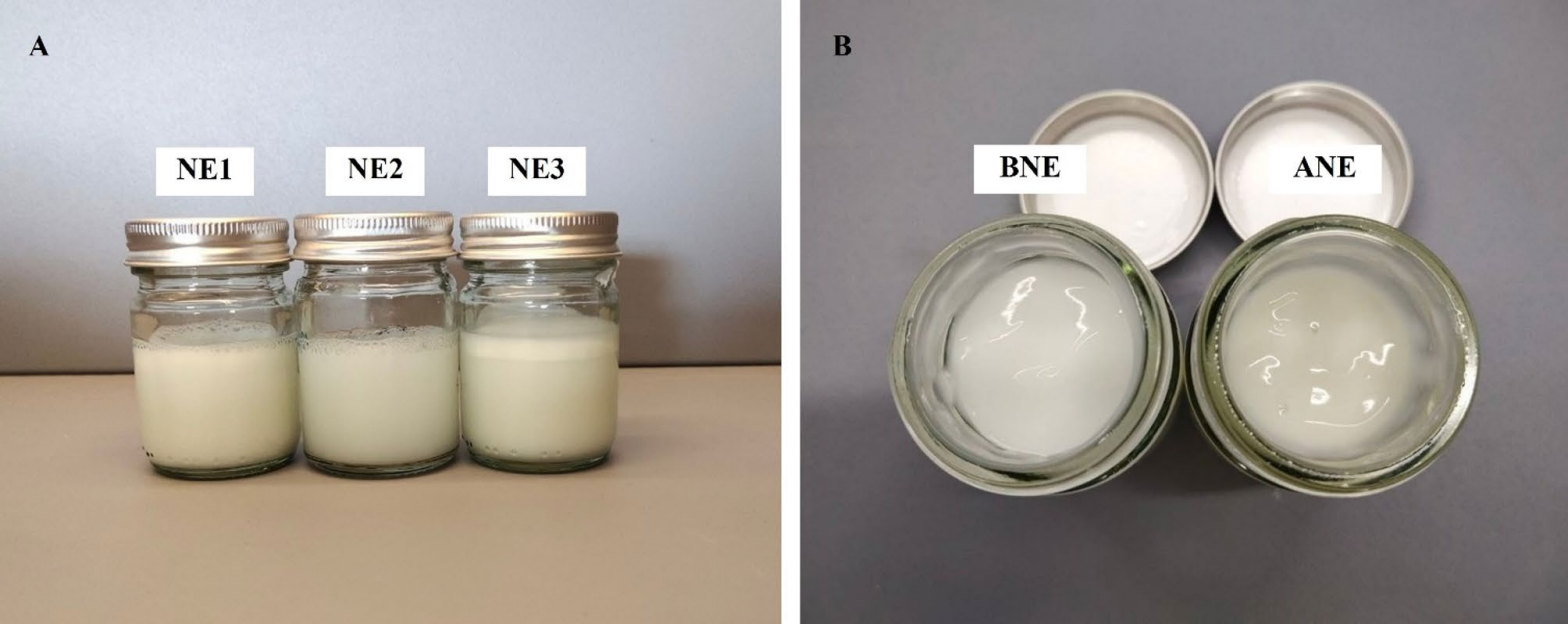
Appearance of (**A**) nanoemulsions (NEs) and (**B**) nanoemulgels (base nanoemulgel (BNE) and avocado extracts-loaded nanoemulgel (ANE).

In visual evaluation of NEs, NE1 and NE2 showed homogeneous NEs, while NE3 showed phase separation (Fig. 5A). The colors of NE1 were opaque; however, NE2 was more translucent. The mean droplet size, PDI, and zeta potential are shown in (Table 4). The droplet size of NE1 (132.46 ± 5.47 nm) was significantly larger than that of NE2 (84.45 ± 5.45 nm). The PDI showed between 0.42 and 0.44. The zeta potential of NE1 and NE2 were − 80.14 ± 0.21 and − 69.94 ± 5.66 mV, respectively. Conversely, parameters for NE3 could not be determined due to phase separation (Table 4).

**Table 4:** The characteristics of nanoemulsions.

Formula	HLB	Droplet size (nm)	Polydisperse index (PDI)	Zeta potential (mV)
NE1	14.9	132.46 ± 5.47	0.44 ± 0.04	−80.14 ± 0.21
NE2	13.3	84.45 ± 5.45	0.42 ± 0.03	−69.94 ± 5.66
NE3	12.2	ND	ND	ND

*ND*  not detectable.

ANE has a light green opaque color while BNE is white opaque (Fig. 5B). ANE and BNE showed no phase separation after preparation. The pH of ANE was 4.75 ± 0.01, showing no significant difference compared to BNE, which had a pH of 4.79 ± 0.03. The viscosity of ANE (41,236.67 ± 72.86 cP) was lower than BNE (51,568.73 ± 1500.93 cP). After heating-cooling cycles, there was no sign of phase separation and no change in the color and pH of the formulations. However, the viscosity of ANE and BNE was significantly increased (Supplementary Table S2).

### Total phenolic content (TPC), in vitro oxidant scavenging activities and non-cytotoxic effects of avocado extracts-loaded nanoemulgel on BJ cells

**Fig. 6:**
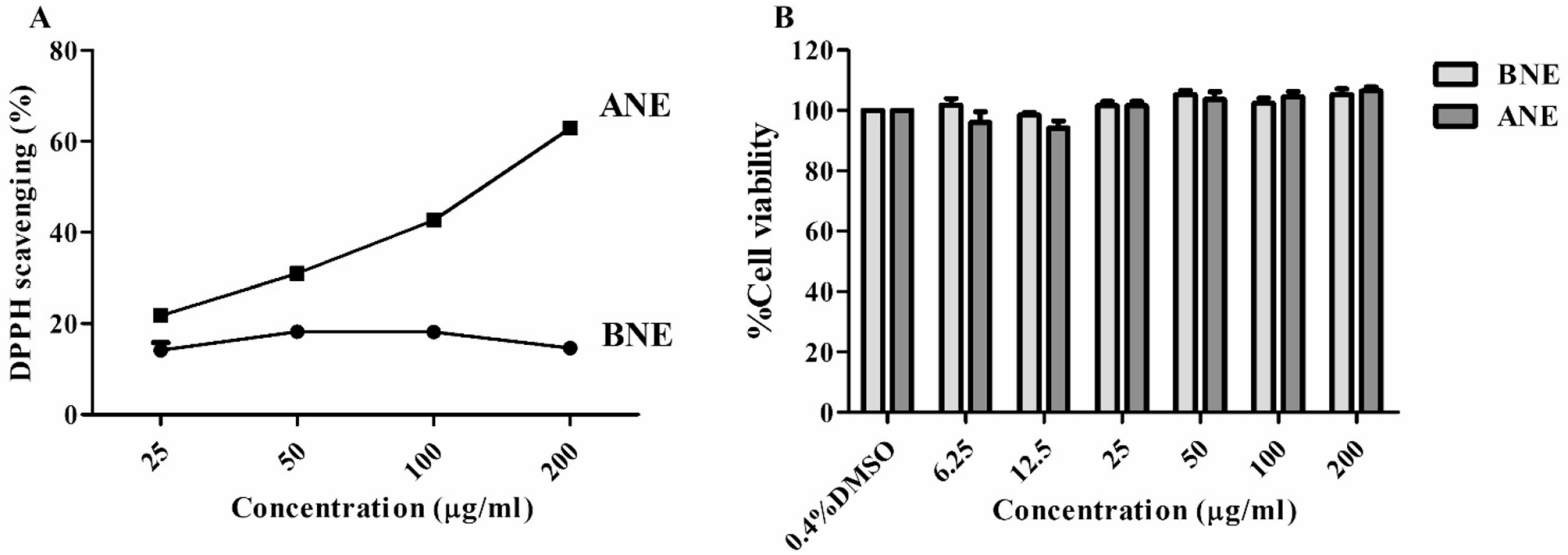
In vitro oxidant scavenging activities and non-cytotoxic effects on BJ fibroblasts of base nanoemulgel (BNE) and avocado extracts-loaded nanoemulgel (ANE). (**A**) Effects of BNE and ANE on DPPH oxidant and (**B**) cell viability of BNE- and ANE-treated BJ cells. The results are presented as the mean ± SEM of three independent experiments. The statistical significance of differences was evaluated by one-way ANOVA followed by Tukey’s test.

TPC of ANE was determined to evaluate the potential biological activity and to support the correlation between the active compound content and antioxidant activity. The result showed that TPC of ANE was 10.81 ± 1.17 mg GAE/g ANE. The ANE was tested for its ability to scavenge the DPPH oxidant. The results revealed that ANE at concentrations of 6.25–200 µg/ml could scavenge the DPPH oxidant by 21.73 ± 0.61–75.84 ± 3.28%, with IC_50_ of 139.59 ± 2.85 µg/ml (Fig. 6A) without cytotoxicity to fibroblast cells by showing the viability of treated cells similar to those of untreated cells (Fig. 6B).

## Discussion

This study investigates the protective effects of avocado peel and seed extracts against UVB-induced skin cell damage. Given that UVB radiation is a main contributor to skin aging and the Earth’s ozone layer is depleted by air pollution, resulting in an increased risk of UVB exposure^34^this research is particularly relevant for developing effective skincare solutions.

UVB radiation is a well-known factor contributing to skin aging, primarily by causing the overproduction of reactive oxygen species (ROS) in fibroblasts, leading to cellular damage^35,36^. Excessive ROS activates several vital pathways, particularly mitogen-activated protein kinases (MAPKs). ROS activates several redox-sensitive signaling pathways of MAPK cascades, including Extracellular signal-regulated kinases (ERK), c-Jun N-terminal kinases (JNK), and p38 MAPK. When ROS oxidize thioredoxin (Trx), a redox-regulating protein, leading to its dissociation from apoptosis signal-regulating kinase 1 (ASK-1). This event triggers the activation of ASK-1, a member of the MAPK kinase kinase (MAPKKK) family, leading to downstream activation of JNK and p38 MAPK pathways^37^. In parallel, ROS can stimulate the ERK pathway by oxidizing sulfhydryl (–SH) groups on cysteine residues of receptor tyrosine kinases (RTKs), such as epidermal growth factor receptors (EGFRs) and platelet-derived growth factor receptors (PDGFRs). This modification promotes the phosphorylation of ERK and its downstream signaling components^38^. Upon UVB irradiation, the ERK pathway plays a key role in the induction of c-Fos, while the p38 and JNK pathways are primarily responsible for activating c-Jun. These two proteins dimerize to form activator protein-1 (AP-1), a transcription factor that regulates the expression of MMPs, including MMP-1, MMP-3 and MMP-9. The upregulation of these MMPs contributes to the degradation of collagen and other extracellular matrix components, thereby accelerating the structural deterioration of the skin associated with photoaging^39^. Among these, MMP-1 plays a critical role in skin aging by degrading type I collagen, the primary structural protein in the dermis, thus contributing to wrinkle formation^40^. Our findings indicate that avocado peel and seed extracts are rich in phenolics (Table 2), which exhibit potent antioxidant properties, as evidenced by their ability to scavenge DPPH and H_2_O_2_ radicals in vitro (Table 3). The results indicate that the antioxidant potential of the extracts was proportional to their total phenolic content, suggesting a positive correlation between phenolic concentration and antioxidant activity. These antioxidant effects were further supported by a significant reduction in UVB-induced ROS overproduction in BJ cells (Fig. 2B). Accordingly, the extracts act as effective antioxidant agents, mitigating MAPK pathway activation, which is upstream of MMP-1 expression. The reduction in oxidative stress was associated with decreased MMP-1 levels (Fig. 3A, B) and enhanced collagen synthesis (Fig. 2C), indicating a protective role against UVB-induced dermal matrix degradation.

In addition, ROS and p38 MAPK are particularly modulated the nuclear factor kappa B (NF-κB)/p65, which further contributes to the regulation of MMPs and pro-inflammatory cytokines, amplifying the inflammatory and degradative processes associated with UVB-induced skin damage^39,40^. UVB exposure has also been shown to upregulate the expression of key pro-inflammatory cytokines, particularly TNF-α, IL-1β, and IL-6, which are key mediators in the photoaging processes^41^. IL-1α and IL-1β promote the degradation of collagen and elastin by upregulating the expression of MMPs^42^. Similarly, IL-6 has been implicated in promoting cellular senescence, a state in which damaged cells permanently stop dividing and secrete pro-inflammatory factors, thereby contributing to tissue dysfunction and accelerating the aging process^43^. Avocado peel and seed extracts demonstrated the ability to reduce the levels of TNF-α, IL-1β, and IL-6 (Fig. 4), suggesting a potential anti-inflammatory effect. This reduction may be associated with the attenuation of reactive oxygen species (ROS), leading to decreased activation of the MAPK and NF-κB signaling pathways. In the future, further investigations may be required to elucidate the underlying molecular mechanisms responsible for these protective effects.

Previous studies have identified various of phenolic compounds in avocado peel and seed extracts, including catechin, epicatechin, caffeic acid, p-coumaric acid, ferulic acid, quercetin-3-O-glucoside, rutin, apigenin, and kaempferol^44^. Among these, catechin has been extensively studied for its antioxidant, anti-inflammatory, and anti-photoaging properties, making it a promising candidate for cosmeceutical applications. Previous studies have demonstrated that catechin can inhibit TNF-α-induced MMP-1 expression, protect against collagen degradation, and reduce intracellular ROS levels in human dermal fibroblasts (NHDFs). These effects are linked to the downregulation of key signaling pathways, including MAPK, Akt, and COX-2, as well as suppression of pro-inflammatory cytokines such as IL-1β and IL-6^45^.

These protective effects of avocado peel and seed extracts collectively contribute to anti-aging benefits by preserving skin integrity, reducing wrinkles, and maintaining skin firmness. Therefore, avocado peel and seed extracts serve as valuable ingredients in the formulation of anti-aging skincare products due to their potent antioxidant and inhibiting MMP-1 and pro-inflammatory cytokine properties. To enhance the delivery and efficacy of bioactive compounds targeting ROS induced MAPK/NFκB/AP-1 pathways, the extracts were formulated into nanoemulgel.

Nanoemulgel is an advanced form of topical delivery system that combines the properties of NEs and gels to enhance the delivery of active compounds to the skin. It represents a promising and innovative approach for the topical delivery of active compounds, offering enhanced penetration, stability, and therapeutic efficacy^46,47^. NEs processing can be prepared by high-energy and low-energy emulsification^48^. High-energy emulsification techniques, including high-pressure homogenization, microfluidization, and ultrasonication, can reduce larger emulsions to nano-sized droplets, thereby controlling droplet size and product stability. However, the heat generated during these processes can accelerate the oxidative degeneration of natural antioxidants^49^. In this study, we formulated NEs using the PIC method to preserve the natural active compounds of avocado extracts and to reduce production costs through a low-energy approach. For o/w NEs, PIC is formed by adding water to the mixture of oil and surfactant/co-surfactant blend. The HLB value of the mixture of surfactant and co-surfactant higher than 10 is a crucial factor for forming Nes^50^. In addition, formulating with both low and high HLB surfactants results in the creation of stable NEs. Consequently, reliable non-ionic surfactants and co-surfactants such as Tween 60 (HLB 14.9) and polyglyceryl-3 diisostearate (HLB 5.5) were selected for the formulation. Even though the HLB of NE1 (14.9), NE2 (13.3), and NE3 (12.2) were more than 10, stable NEs were formed only in NE1 and NE2. The particle size, PDI, and zeta potential are vital indexes that characterize NEs’ uniformity, dispersibility, and stability^51^. The observed larger droplet size in NE1 compared to NE2 may be attributed to the fact that surfactant alone might not adequately reduce the interfacial tension between oil and water, as opposed to when surfactant is combined with a co-surfactant^52^. Another factor is that NE1 has a higher oil content in its formulation compared to NE2. This higher oil content leads to increased saturation of emulsifiers, resulting in larger droplet sizes^53^. Additionally, these contribute to an increase in PDI. Nevertheless, with its larger tail volume, the abundance of polyglyceryl-3 diisostearate in NE3 could potentially permit excessive oil penetration into the hydrocarbon tail, leading to phase separation. The negative charge of droplet surfaces as zeta potential values of NE1 and NE2 were displayed in the range of -69 to -81 mV, indicating the high repulsive force between nanodroplets and preventing the chance of coalescence in the system. As the physicochemical characteristics, NE2 was suitable for developing nanoemulgel.

Transforming NEs into nanoemulgel has extended their duration on the skin, increasing deposition percentages over time. Additionally, the permeation of drugs into the skin is highest in nanoemulgel when compared to emulgel, gel, and suspension forms^54^. The topical application of nanoemulgel has been reported to have a higher efficacy treatment, such as curcumin and resveratrol loaded in nanoemulgel revealed decreased inflammatory mediator levels and efficient enhancement in wound healing^55,56^.

In this study, we incorporated a water-soluble cross-link acrylic polymer, Aqupec HV-505E, into the NEs system to provide the nanoemulgel. An effective dose of avocado peel and seed extracts at 5% w/w (dissolved in propylene glycol) was incorporated into nanoemulgel, namely ANE. This formulation showed good physicochemical characteristics, such as color, pH, and viscosity, which are suitable for skin use. This study showed that avocado peel and seed extracts and ANE revealed potentially reduce the DPPH oxidant as well. These results suggest that the avocado extracts incorporated in nanoemulgel may be protected from oxidative processes. This protection occurs due to the NEs process, which prevents optimal contact between antioxidant compounds and free radicals. In NEs, the bioactive compounds are shielded by the lipid layer that forms the droplets^57^. Due to the small droplet size, NEs provides a larger surface area and better interaction with the skin surface, thereby facilitating increased drug concentration permeation and bioavailability of active compounds at the target site^51^. Therefore, the avocado phenolic compounds encapsulated within the small oil droplets of the NE2, represented in ANE, can effectively penetrate the skin, thereby enhance cellular antioxidant activity and exert anti-photoaging effects observed in vitro. The slightly lower pH of ANE compared to BNE could be potentially attributed to phenolic compounds present in the avocado peel and seed, such as gallic acid, caffeic acid, hydroxybenzoic acid, coumaric acid, and quinic acid^58,59^. According to previous studies, phenolic compounds are stable in acidic conditions (pH < 7)^60^. Thus, the acidic nature of ANE (pH 4.7) contributes to the stabilization of phenolic compounds, which in turn enhances the overall efficacy of the product. The higher viscosity of nanoemulgel compared to NEs provides improved topical application, increased retention at the application site, and enhanced skin permeation. According to previous research, *Kunzea ericoides* nanoemulgel significantly enhances sustained drug release and provides prolonged therapeutic effects compared to *K. ericoides* nanoemulsion and solution forms^61^. Metformin-loaded nanoemulgel demonstrated sustained drug release and enhanced anti-aging effects in vitro, ex vivo, and in vivo, as evidenced by reduced MMP-1 expression, improved dermal collagen, and reduced wrinkles compared to conventional treatments^62^. Therefore, the suitable viscosity of ANE may facilitate the phenolic compounds with a prolonged effect and enhanced therapeutic efficacy.

The stability testing revealed that the pH of ANE remained stable, which is a positive indicator of its formulation integrity. The observed changes in viscosity after the stability test may be attributed to the low pH of the formulations (pH 4.7), which is below the optimal range (6.5–7.5) required for complete gel formation of carbomer^63^. Additionally, the presence of propylene glycol may have slowed the swelling process by reducing water availability and hindering solvent diffusion into the polymer network^64,65^. As a result, incomplete gel formation during the heating–cooling cycle likely led to a gradual increase in viscosity due to continued structural development over time.

To evaluate the biological activities of ANE, TPC, antioxidant activity using the DPPH assay, and cytotoxicity assays were conducted. Notably, ANE maintained a high TPC and exhibited strong antioxidant activity, while demonstrating no cytotoxic effects on skin cells. These findings suggest that ANE is a promising candidate for skin applications, particularly in anti-photoaging formulations.

Although the present study provides promising findings, certain limitations may be addressed in further research, including the quantification of active compounds in the avocado extracts to ensure quality control and study release and skin permeation studies. These are to ensure that the developed formulation possesses anti-photoaging efficacy, safety, and physicochemical stability.

## Conclusions

Avocado peels and seeds are the remnant parts of this edible fruit. Their ethanolic extracts reveal potential antioxidant properties by scavenging in vitro free radicals, DPPH, and H_2_O_2_ and protecting against UVB-damaged fibroblasts by reducing intracellular ROS in UVB-irradiated cells. The avocado peel and seed extracts also reduce UVB-activated MMP-1 expression and pro-inflammatory cytokine production in fibroblasts, leading to an increase in type I collagen, the main extracellular matrix maintaining the strength and elasticity of the skin. Our studies provided beneficial effects of avocado peel and seed extracts as active ingredients in cosmeceutical anti-photoaging product. Next, the avocado peel and seed extracts are formulated into nanoemulgel, an effective formulation that increases potency for anti-skin aging. The avocado extracts-loaded nanoemulgel has good characteristics for use on the skin, still has antioxidant properties, and is non-cytotoxic to skin cells. These properties of the formulation are advantageous as a novel cosmeceutical anti-photoaging product. However, further research is warranted to address certain limitations and to comprehensively ensure the formulation’s efficacy, safety, and stability.

## Supplementary Information

Below is the link to the electronic supplementary material.

Supplementary Material 1

## Data Availability

Availability of data and materialsMaterials and data associated to this study are available from the corresponding author upon request.
