# Supplementary material for: Protective effects of avocado peel and seed extracts against UVB-damaged fibroblasts for the development of an anti-photoaging nanoemulgel
Source: Sci Rep. 2025 Jul 31;15:28006. doi: 10.1038/s41598-025-13679-9 (PMC12314098; doi:10.1038/s41598-025-13679-9)
Supplement: Supplementary file 1 — Supplementary Material 1 [file 41598_2025_13679_MOESM1_ESM.pdf]

Supplement information

Protective effects of avocado peel and seed extracts against UVB-damaged fibroblasts for the development of an anti-photoaging nanoemulgel

Suradwadee Thungmungmee[1] and Nakuntwalai Wisidsri[1,*]

[1] Faculty of Integrative Medicine, Rajamangala University of Technology Thanyaburi, Pathum Thani, 12130, Thailand. [*]Email: nakuntwalai_w@rmutt.ac.th

[*]Corresponding author: Nakuntwalai Wisidsri, Tel +66629194515, E-mail address: nakuntwalai_w@rmutt.ac.th

Table of contents

		Page
Table S1	Composition of nanoemulsions	2
Table S2	Physicochemical characteristics of nanoemulgels	2
Figure S1	The original Western blots of presenting in Figure 3A	3
Figure S2	The original Western blots of presenting in Figure 3B	9

Table S1 Composition of nanoemulsions

Ingredients	INCI name	Function	NE1	NE2	NE3
Tween 60	Polysorbate 60	Surfactant	10.00	10.00	10.00
Polyglyceryl-3 diisostearate	Polyglyceryl-3 diisostearate	Cosurfactant	-	2	4
avocado oil:isononyl isononanoate (1:10)	*Persea americana* (avocado) oil:isononyl isononanoate	Emollient	10.00	10.00	10.00
Deionized water	Aqua	Solvent	80.00	78.00	76.00

Table S2 Physicochemical characteristics of nanoemulgels

Formula	Heating-cooling 7 cycles							
	Color		Phase separation		pH		Viscosity (cP)	
	Before	After	Before	After	Before	After	Before	After
Base nanoemulgel (BNE)	White opaque	White opaque	no	no	4.79±0.03	4.79±0.02	51568.73 ±1500.93	60370.56±477.32*
Avocado extract-loaded nanoemulgel (ANE)	Light green opaque	Light green opaque	no	no	4.75±0.01	4.76±0.00	41236.67±72.86	50533.33±635.63*

*$p < 0.05$ denotes a statistically significant difference when compared to the pre-stability test.

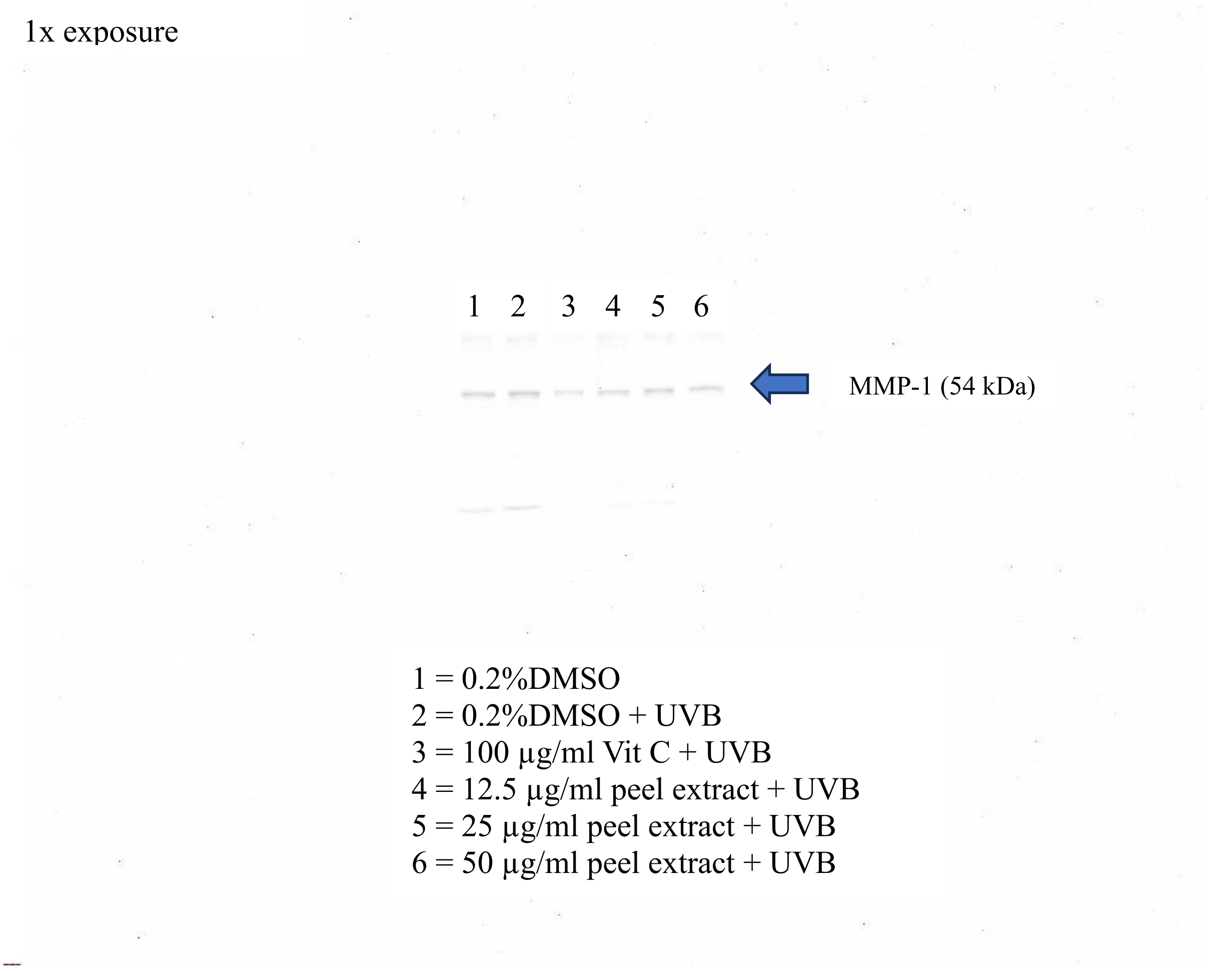

Figure S1 The MMP-1 original Western blots of presenting in Figure 3A

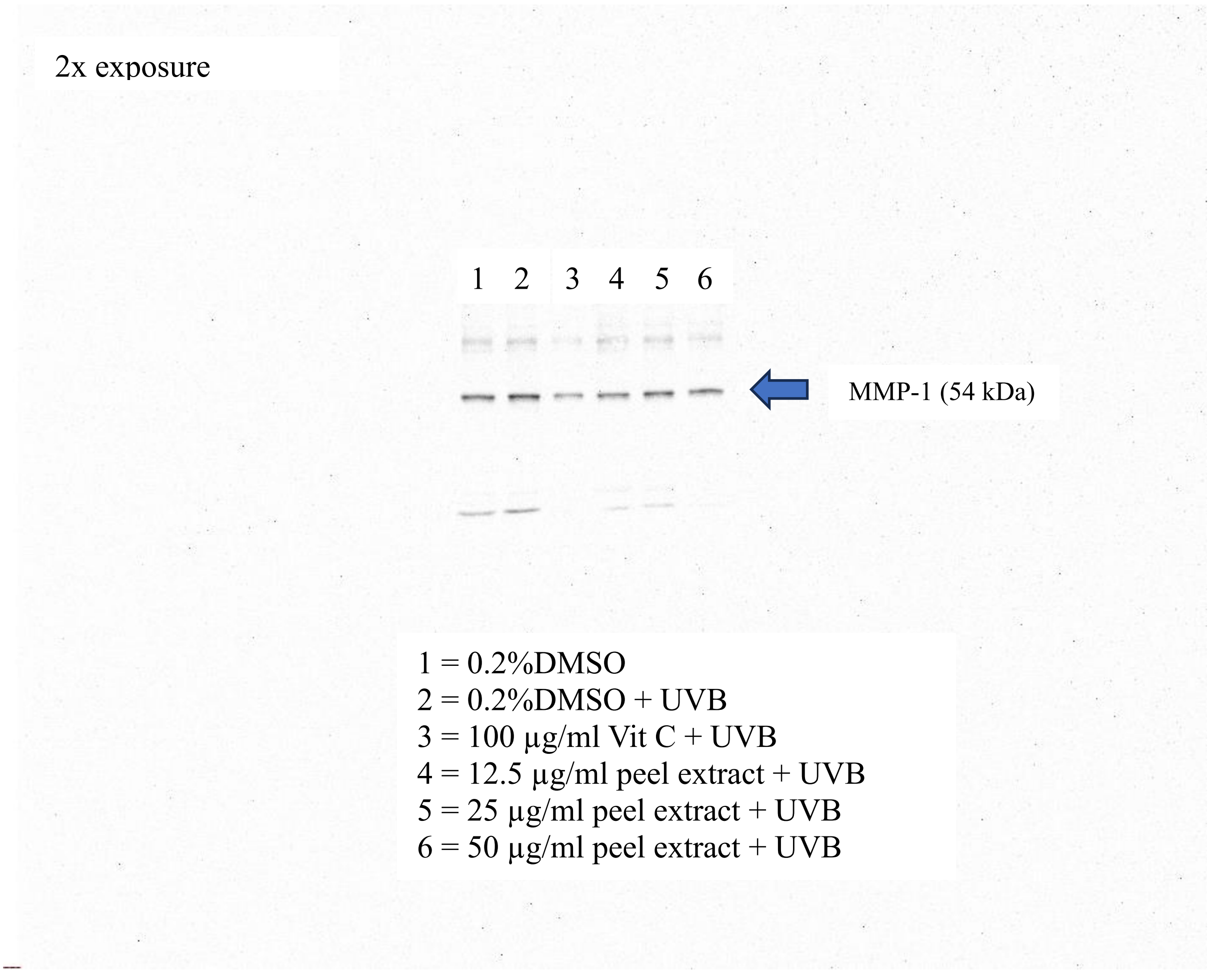

Figure S1 (continue) The MMP-1 original Western blots of presenting in Figure 3A

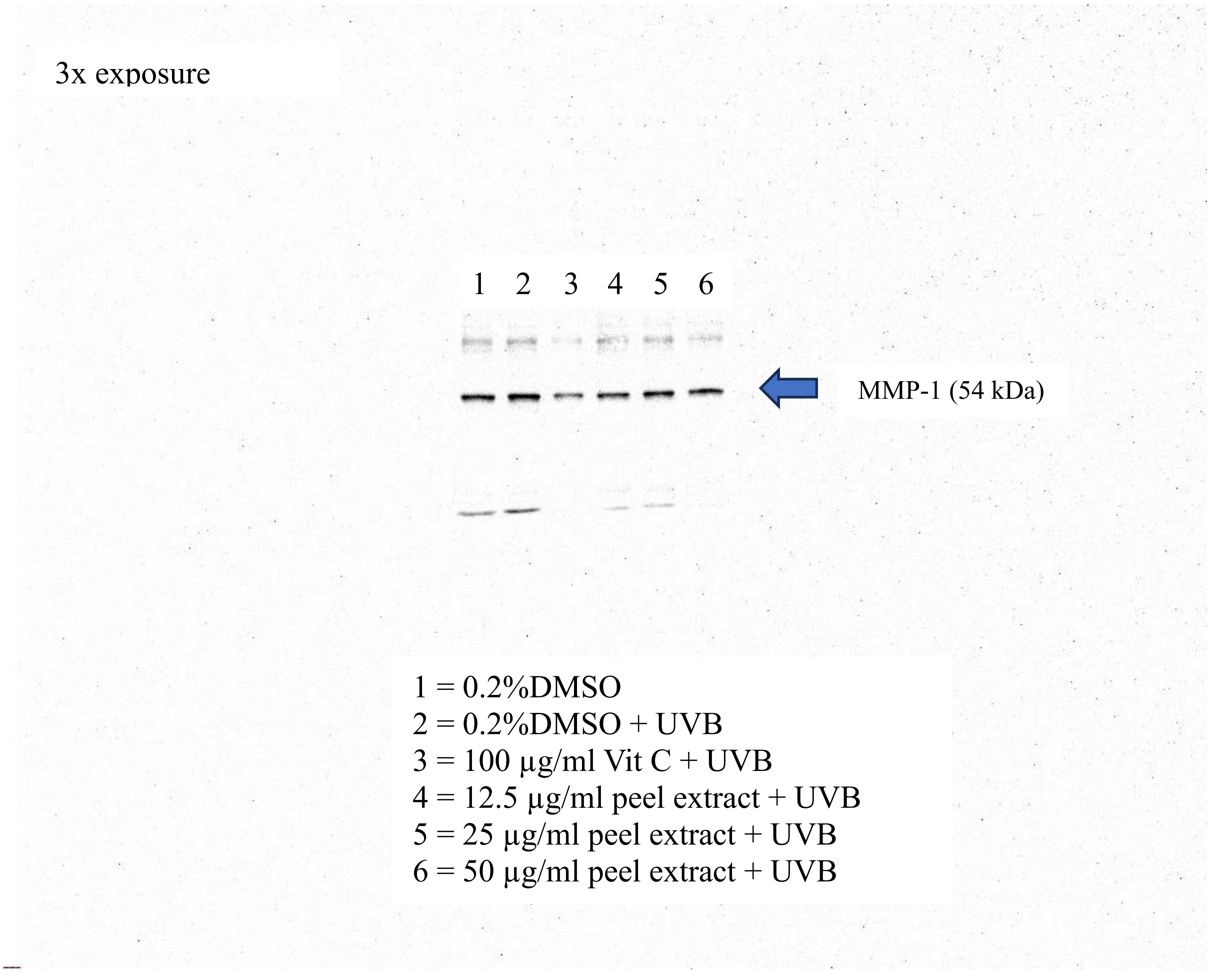

Figure S1 (continue) The MMP-1 original Western blots of presenting in Figure 3A

1x exposure

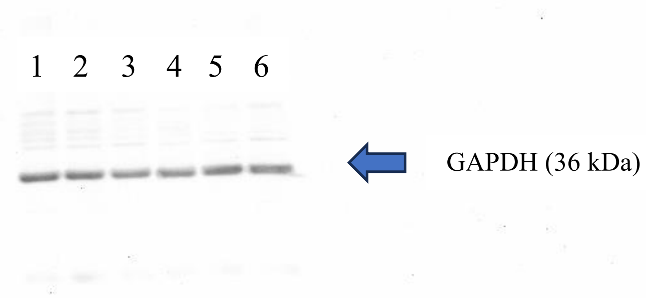

1 = 0.2%DMSO
2 = 0.2%DMSO + UVB
3 = 100 μg/ml Vit C + UVB
4 = 12.5 μg/ml peel extract + UVB
5 = 25 μg/ml peel extract + UVB
6 = 50 μg/ml peel extract + UVB

Figure S1 (continue) The GAPDH original Western blots of presenting in Figure 3A

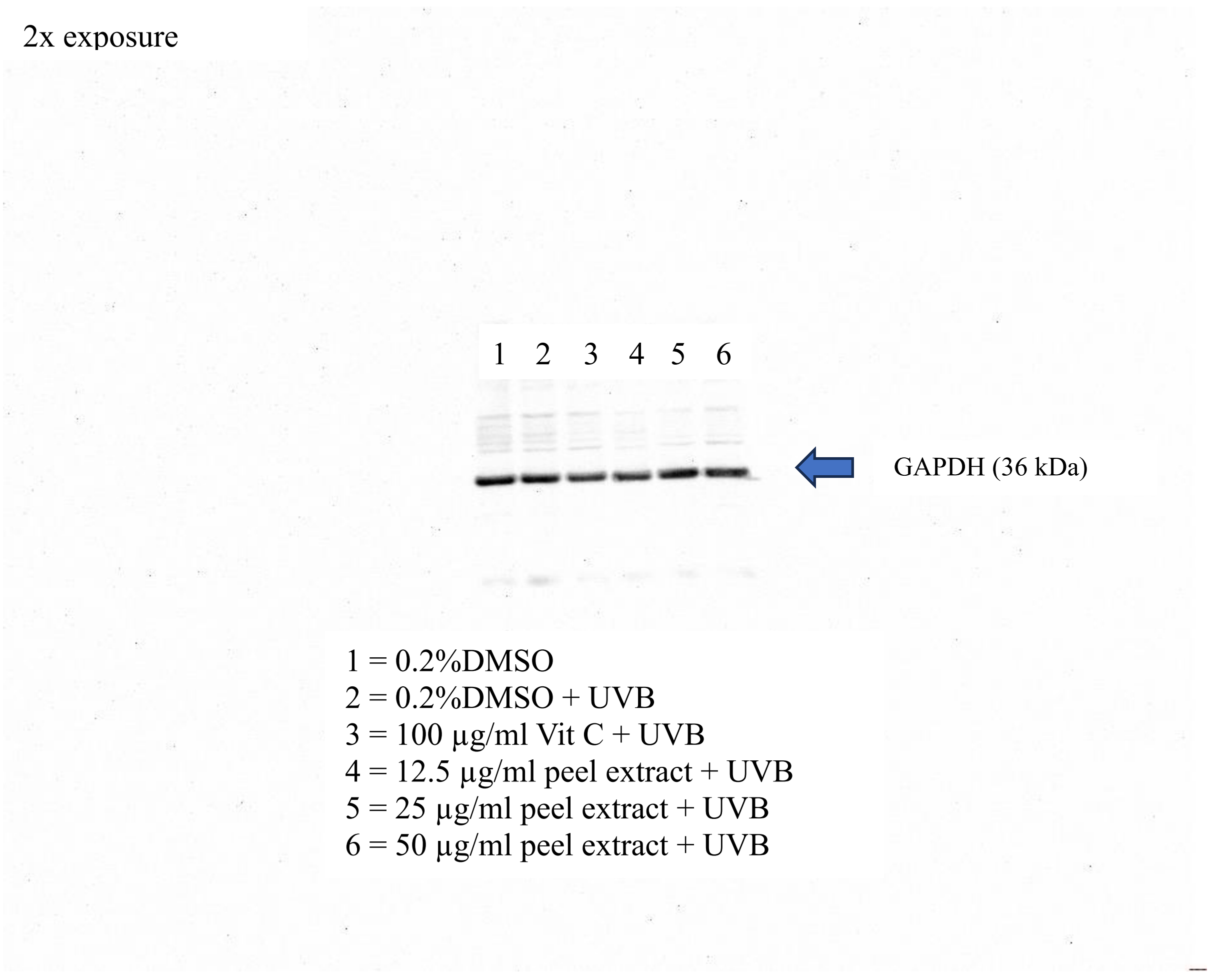

Figure S1 (continue) The GAPDH original Western blots of presenting in Figure 3A

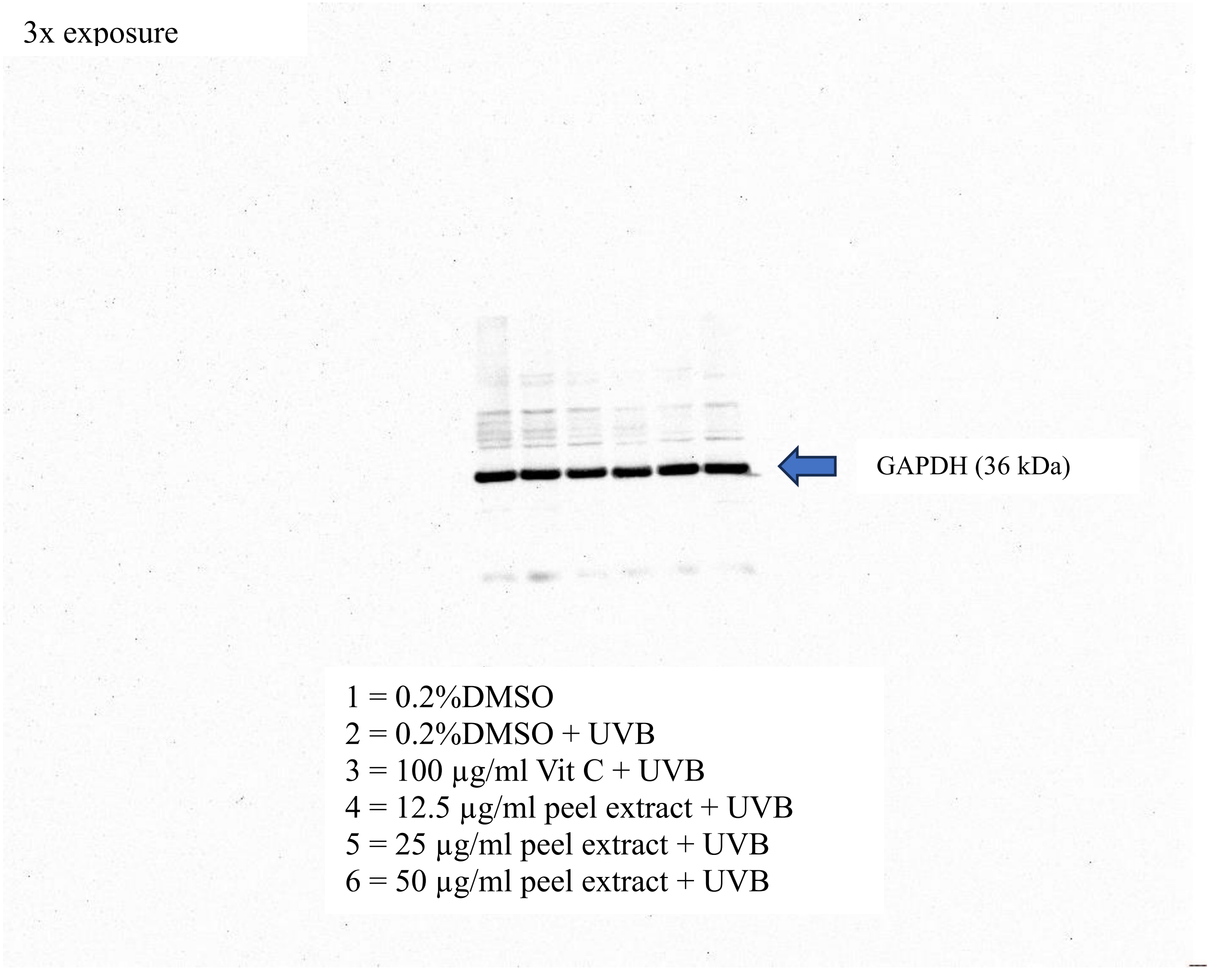

Figure S1 (continue) The GAPDH original Western blots of presenting in Figure 3A

1x exposure

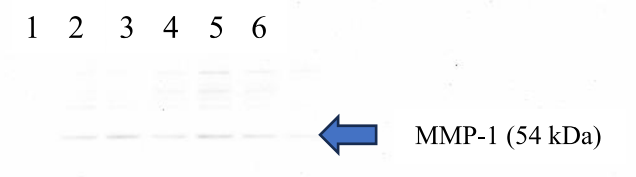

1 = 0.2%DMSO
2 = 0.2%DMSO + UVB
3 = 100 µg/ml Vit C + UVB
4 = 12.5 µg/ml seed extract + UVB
5 = 25 µg/ml seed extract + UVB
6 = 50 µg/ml seed extract + UVB

Figure S2 The MMP-1 original Western blots of presenting in Figure 3B.

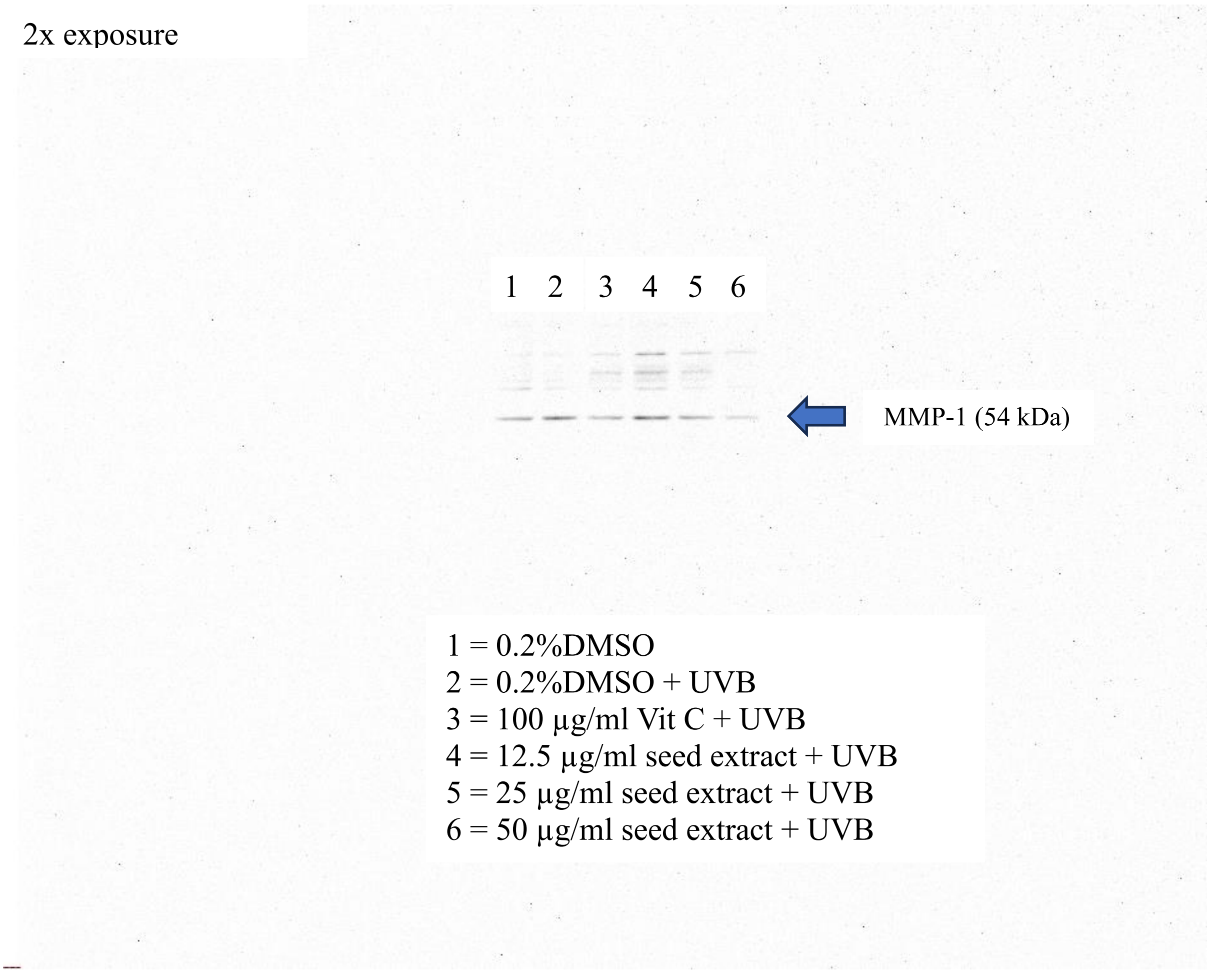

Figure S2 (continue) The MMP-1 original Western blots of presenting in Figure 3B.

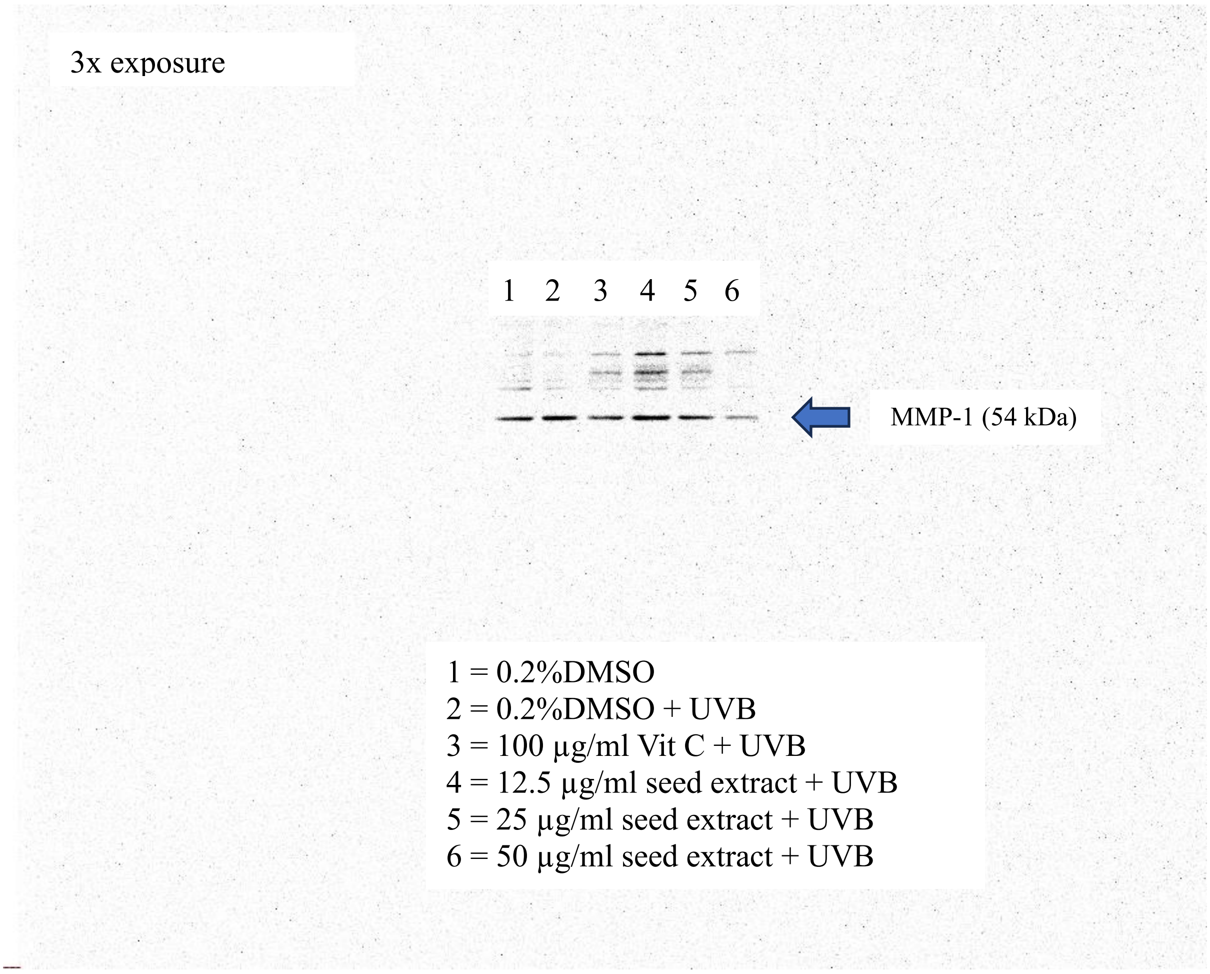

Figure S2 (continue) The MMP-1 original Western blots of presenting in Figure 3B.

1x exposure

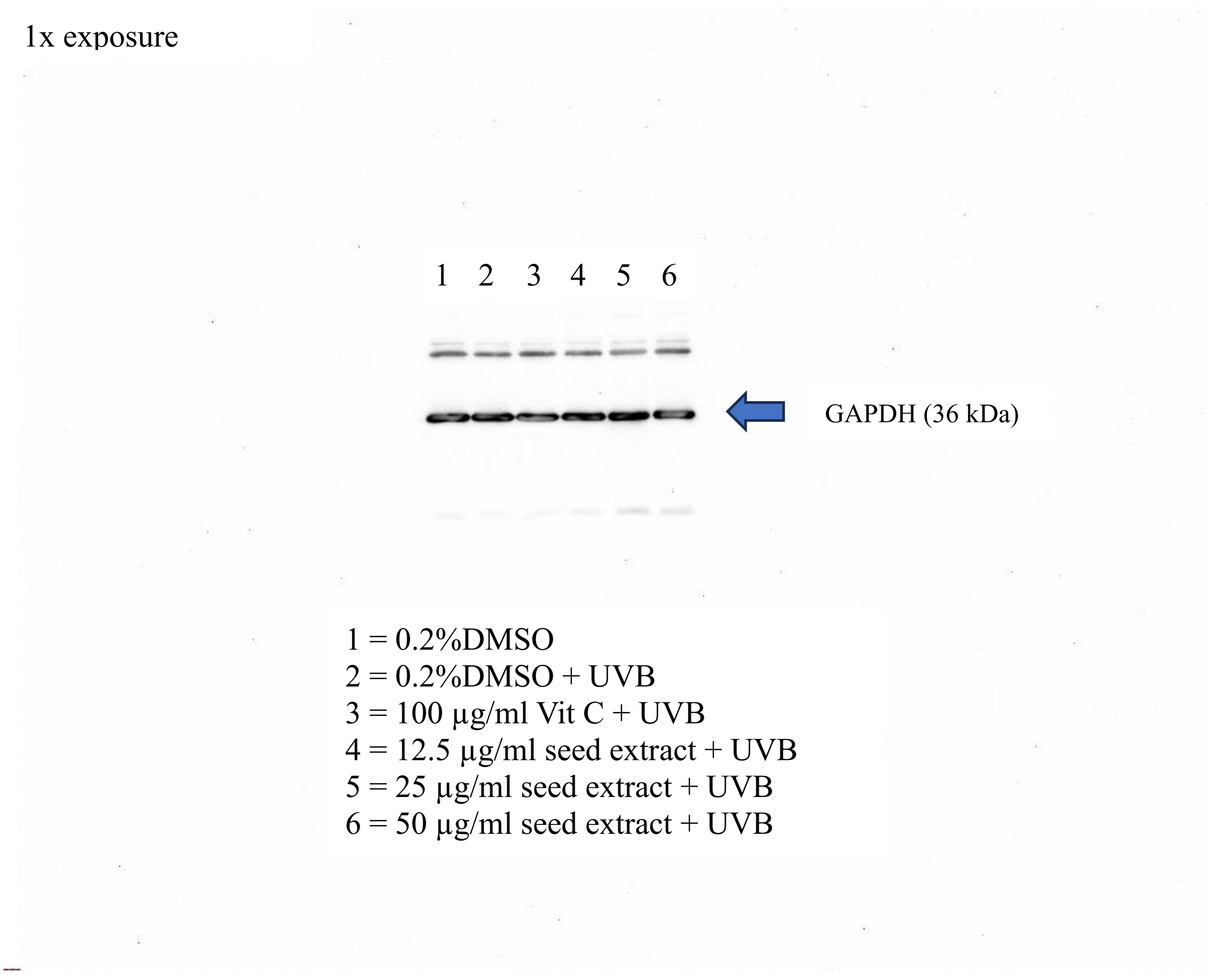

Figure S1 (continue) The GAPDH original Western blots of presenting in Figure 3B

2x exposure

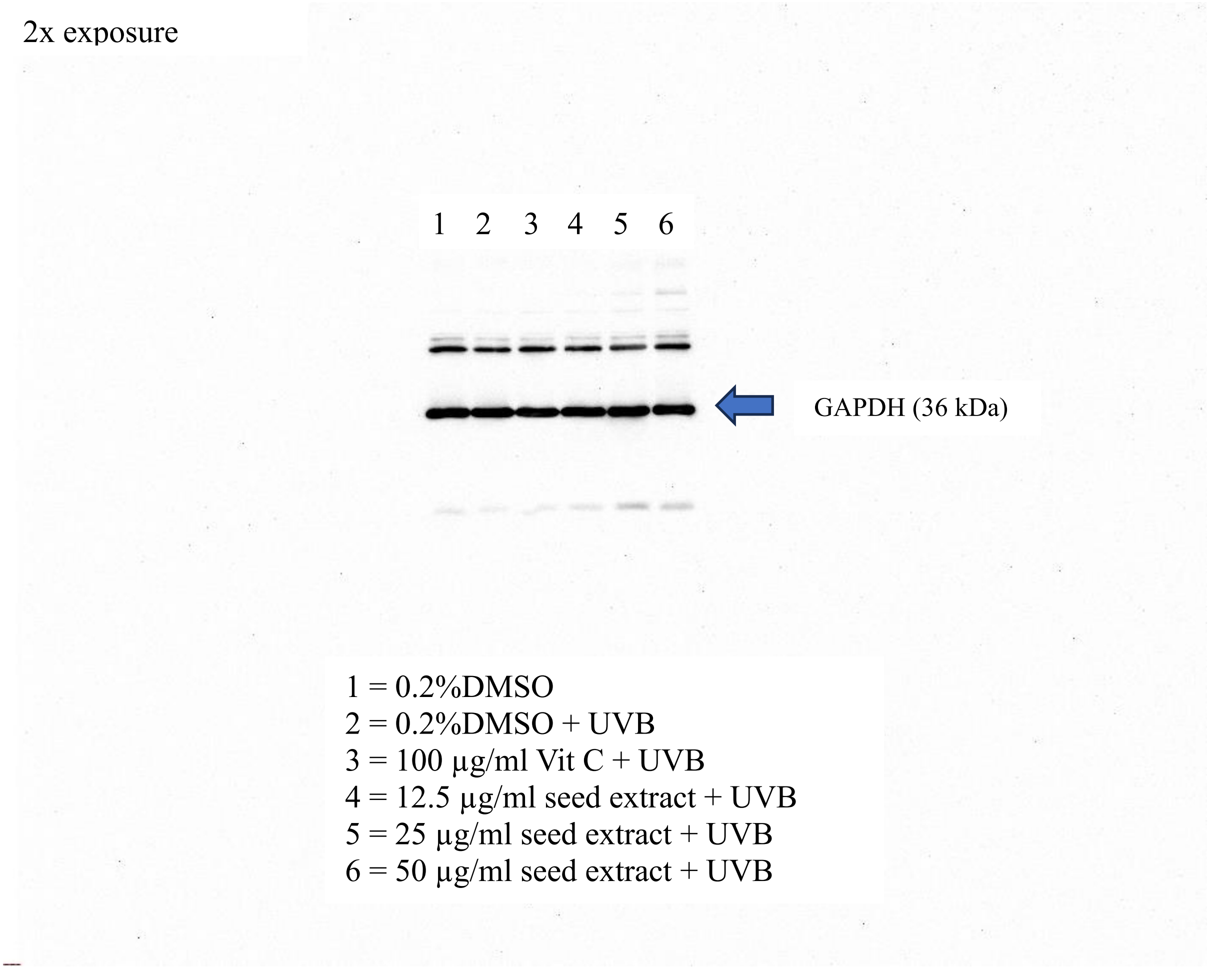

Figure S1 (continue) The GAPDH original Western blots of presenting in Figure 3B
